# Letter From the Editor

**DOI:** 10.5334/pb.1123

**Published:** 2021-09-28

**Authors:** Steve Majerus

**Affiliations:** 1 Psychology & Neuroscience of Cognition Research Unit, Université de Liège, Belgium; 2 Fund for Scientific Research FNRS, Belgium

**Keywords:** editorial, peer review, editorial board, open access, data availability

During my second term as Editor-in-Chief of our journal Psychologica Belgica, I have the pleasure to highlight a number of major events for our journal. First, we have increased the editorial board to 17 associate editors (*https://www.psychologicabelgica.com/about/editorialteam/*) for handling the peer review process and the increasing number of submissions. I thank all the associate editors for their time and expertise they invest in making our journal publish most rigorous scientific research. I also take this opportunity to thank all the reviewers for the quality of their reviewing, a selfless activity which can be time-consuming but which is essential for maintaining the theoretical and methodological standards of a scientific journal.

Second, and thanks to the work of our editors, reviewers and authors, the metrics of our journal reveal a steadily rising impact of the articles that are published. The 2020 impact factor is 1.52 (up from 0.43 five years ago) and the current 5-year impact factor is 2.38 (Clarivate®). It is also important to stress that manuscript rejection rates exceed 50%. As already noted, the number of papers published is increasing, with 22 papers published in 2019, 24 in 2020 and already 20 for the first half of 2021.

Third, our journal has been able to reach these numbers despite, or perhaps because, of the Covid-19 pandemic situation. In 2020, we launched a special issue on the psychological factors associated with the Covid-19 situation. This special issue has attracted a particularly large number of submissions, six of which have already been published. The call is now closed and the continuous publication scheme of our journal allows us to publish articles immediately after the completion of the peer review process, thus authors do no need to wait until the entire special issue is compiled before seeing their work getting disseminated. This editorial policy is particularly important for the publication of research characterized by time-sensitive issues such as the new psychological challenges caused by the Covid-19 pandemic.

Fourth, our journal will soon be able to benefit from a new and optimized manuscript handling platform, to the great benefit of our authors, editors and reviewers for the submission, reviewing and editing of the different types of articles we accept. Psychologica Belgica, a flagship publication of the Belgian Association of Psychological Sciences (BAPS), publishes standard research reports, but also theoretical reviews and statistical notes. I also remind here that Psychologica Belgica is a full open access journal with minimal publication fees (and of which regular BAPS members are exempted). Truly open access does not only concern a scientific paper but also the data from which it is derived. Therefore, Psychologica Belgica encourages that all anonymized data are fully and freely accessible, an encouragement that will be transformed into a requirement during my second and last term as Editor-in-Chief (see also Majerus, 2018).

In closing, I would like to express my gratitude to Prof. Laurence Claes, whose term as an associate editor came to an end. She has been a long-standing member of our editorial board and spent countless hours in editing manuscripts, chasing reviewers and writing decision letters. We thank you for your professionalism and devotion to Psychologica Belgica. I also reiterate all my thanks to the associate editors who remain on board and to those who have newly joined our team.
